# Assessment of mineral intake in the diets of Polish postmenopausal women in relation to their BMI—the RAC-OST-POL study: Mineral intake in relation to BMI

**DOI:** 10.1186/s41043-016-0061-1

**Published:** 2016-08-02

**Authors:** Dominika Głąbska, Dariusz Włodarek, Aleksandra Kołota, Aleksandra Czekajło, Bogna Drozdzowska, Wojciech Pluskiewicz

**Affiliations:** 1 Department of Dietetics, Faculty of Human Nutrition and Consumer Sciences, Warsaw University of Life Sciences-SGGW, 159c Nowoursynowska Street, 02-776 Warsaw, Poland; 2 Department of Nephrology, Regional Hospital in Racibórz, Racibórz, Poland; 3 Department of Pathomorphology, Medical University of Silesia, Katowice, Poland; 4 Metabolic Bone Diseases Unit, Department and Clinic of Internal Diseases, Diabetology and Nephrology, Medical University of Silesia, Katowice, Poland

**Keywords:** BMI, Intake, Minerals, Postmenopausal women

## Abstract

**Background:**

The diets of postmenopausal women in Western countries tend to be deficient in minerals, even if the energy value is at the recommended level. The objective of the presented population-based cohort study was to assess the intake of minerals (sodium, potassium, calcium, phosphorus, magnesium, iron, zinc and copper) in the diets of women aged above 55 years and to analyse the relations between BMI and mineral intake in this group.

**Methods:**

The study was conducted in a group of 406 women who were randomly recruited from the general population of those aged above 55 years. The main outcome measures included BMI, reported sodium, potassium, calcium, phosphorus, magnesium, iron, zinc and copper intake assessed by dietary record (conducted during two typical, non-consecutive days). The distribution was verified with the use of the Shapiro-Wilk test. The comparison between groups was conducted using ANOVA with the LSD post hoc test or Kruskal-Wallis ANOVA with multiple comparisons. A comparison of satisfying nutritional needs was conducted using the chi-square test.

**Results:**

Normal body weight individuals were characterised by lower sodium intake per 1000 kcal of diet than obese class II and III individuals (BMI ≥ 35.0 kg/m^2^). Overweight individuals were characterised by lower potassium and magnesium intake per 1000 kcal of diet than obese class I individuals (BMIϵ < 30.0; 35.0 kg/m^2^). The majority of individuals was characterised by insufficient potassium, calcium and magnesium intake. No differences in satisfying nutritional needs between BMI groups were observed for all minerals.

**Conclusions:**

Following an improperly balanced diet was observed in the group of postmenopausal female individuals analysed. It was stated that the daily intake of all the assessed minerals was not BMI-dependent for the postmenopausal female individuals, but the nutrient density of diet (for sodium, potassium and magnesium) was associated with BMI.

## Background

The diets of postmenopausal women in Western countries generally tend to be improperly balanced. Female subjects are characterised by a too low intake of vitamins, especially of vitamins D and E [1], as well as of B vitamins [2]. Their diets are also deficient in minerals, especially in calcium [3], magnesium [4], iron [5], zinc [6] and iodine [7]. The above-mentioned low intake of specific nutrients is often observed even if the energy value of the diet is proper [8, 9].

Simultaneously, the results of the analysis encompassing Polish postmenopausal women indicate insufficient energy intake [10] and energy intake decreasing over the years [11], even for overweight and obese individuals [12]. The results from other Western countries are similar [13–15]. However, the low energy intake, obtained from the dietary record or food frequency questionnaire, may be associated with the limitations of the above-mentioned methods [16]. This may result from the fact that individuals tend to underestimate the quantity of consumed food products, which is observed especially in patients with an increased amount of body fat [17], with chronic diseases [18], a lower education level [19], those dissatisfied with their body image [20] and motivated to follow a proper diet [21].

As a consequence, the conclusions associated with nutrient intake, especially the macronutrients and energy value of the diet, must be prudential and balanced [22]. This is even reflected in the Polish recommendations to assess the energy value of the diet, where it is specified that the energy value of the diet in groups of individuals should not be assessed on the basis of values calculated from the dietary record or food frequency questionnaire but rather on the basis of BMI [23]. If individuals have a recommended BMI, their intake is constant and the body mass does not change, it may be interpreted that the energy intake is at the recommended level.

The aim of the study was to assess the intake of minerals (sodium, potassium, calcium, phosphorus, magnesium, iron, zinc and copper) in the diets of Polish women aged above 55 years in comparison with the recommendations and to analyse relations between BMI and mineral intake in this group (RAC-OST-POL study data).

## Methods

The RAC-OST-POL study was conducted in a random group of Polish women aged above 55 years who were recruited from the general population of women aged above 55 years in the District of Raciborz, in the south of Poland. The age group above 55 years old was randomly chosen on the basis of guidelines on standard international age classifications by the United Nations Organization based on World Health Organization classification [24]. Of all the women inhabiting the region who were above 55 years old at the time of enrolment, 10 % were randomly chosen on the basis of their personal identity numbers (1750 individuals) and invited to participate in the study, which was conducted in May 2010. This study was conducted according to the guidelines laid down in the Declaration of Helsinki, and all procedures involving human subjects were approved by the Ethics Committee of the Medical University of Silesia, Katowice, Poland. A written informed consent was obtained from all participants.

A group of 979 individuals responded positively to the invitation, and of these, a total of 405 individuals completed a 2-day dietary record during the whole following month (no dietary records were rejected during the analysis). Body weight and height were measured with a standard medical balance and used to calculate the BMI (kg/m^2^). On the basis of the conducted interview, the physical activity level in the assessed group was stated to be low-to-moderate and stable body mass condition was declared by the vast majority of individuals for the previous 3 months.

The assessment of diet was based on self-reported data from the patients’ 2-day dietary record conducted on random days. Such record was also applied in the Nationwide Food Consumption Survey [25]. Two random days were chosen, as the analysed women in general were not working professionally, and in the research of other authors [26], no differences between weekdays and days of the weekend were proved in female individuals who were not working professionally, as their diet on weekdays and days of the weekend did not differ. Participants were asked to conduct a dietary record during two typical, non-consecutive days. To provide reliable estimates of food intake, the participants were instructed about the principles of conducting a dietary record and about the necessity of accurate and scrupulous recording of all food products consumed and beverages drunk. If they were not able to conduct a weighted food record, they were asked to conduct an estimated food record (using estimated serving sizes).

The results were analysed by using Polish dietician software, “Dietetyk 2,” and the Polish database of the nutritional values of products [27], with additional label information collected by the participants, while sodium chloride addition during the preparation of meals was not taken into account. The obtained average nutritional value of the analysed diets (mean of two analysed days) was the basis for further analysis.

The mineral contents in the diets were compared with the recommendations for healthy women (Table 1); for calcium, phosphorus, magnesium, iron, zinc and copper at the Estimated Average Requirement (EAR) level; and for sodium and potassium at the Adequate Intake (AI) level because the EAR level had not been established [28]. In a previously conducted assessment of the diet of women being analysed, it was concluded that a number of women were unable to satisfy their calcium requirement exclusively from their diet [29].

**Table 1:** Patients’ characteristics, accompanied by comparison between BMI groups, RAC-OST-POL study, May 2010

Group	Calculated value	Age (years)	Weight (kg)	Height (cm)	BMI (kg/m^2^)
Total *N* = 405	Mean	66.5	74.3	155.9	30.6
	SD	7.9	13.6	5.8	5.4
	Median	65.3^†^	73.0^†^	156.0	30.0^†^
	Minimum	55.0	44.0	136.0	19.7
	Maximum	92.2	120.0	171.0	47.4
BMIϵ <18.5; 25.0 kg/m^2^, *N* = 61	Mean	63.5	57.1	157.0	23.1
	SD	6.7	5.6	6.5	1.5
	Median	61.8^†a^	58.0^a^	157.0	23.5^†a^
	Minimum	55.2	44.0	141.0	19.7
	Maximum	79.5	68.0	171.0	25.0
BMIϵ <25.0; 30.0 kg/m^2^, *N* = 140	Mean	66.3	67.4	156.4	27.5
	SD	8.2	5.9	5.4	1.4
	Median	64.5^†ab^	67.0^b^	156.0	27.3^†b^
	Minimum	55.0	52.0	141.0	25.1
	Maximum	92.2	83.0	170.0	30.0
BMIϵ <30.0; 35.0 kg/m^2^, *N* = 118	Mean	67.3	78.5	155.5	32.4
	SD	7.8	7.0	6.1	1.4
	Median	67.8^†b^	78.0^c^	156.0	32.4^†c^
	Minimum	55.1	63.0	136.0	30.0
	Maximum	84.1	95.0	171.0	34.9
BMI ≥ 35.0 kg/m^2^, *N* = 86	Mean	67.7	92.2	155.0	38.4
	SD	7.7	9.9	5.3	2.9
	Median	67.3^†b^	92.0^d^	155.0	37.6^†d^
	Minimum	55.1	71.0	139.0	35.0
	Maximum	89.0	120.0	166.0	47.4
*p* ^e^	*p* ^e^	0.0057	0.0000	0.1299	0.0000

^†^Variable was not normally distributed (verified by the Shapiro-Wilk test; *p* < 0.05)

^a, b, c, d^Mean/median values within the column with unlike superscript letters were significantly different (*p* < 0.05)

^e^Differences assessed by the ANOVA/Kruskal-Wallis ANOVA

Statistical analysis was conducted using Statistica software version 8.0 (StatSoft Inc.) and Statgraphics Plus for Windows 4.0. The distribution of the analysed factors was verified by using the Shapiro-Wilk test. The comparison of mineral intake between groups was conducted using ANOVA accompanied by the LSD post hoc test as well as, in the case of distribution that was different than normal, using Kruskal-Wallis ANOVA accompanied by multiple comparisons. The median values are presented in the figures. A comparison of satisfying nutritional needs was conducted using the chi-square test. An additional statistical analysis (chi-square test) was performed in order to confirm that the analysed population could be treated as a representative subsample—it was verified whether the mean age in the analysed subpopulation matched the mean age in the general population. The two-sided level of significance *p* ≤ 0.05 was accepted.

## Results

Table 1 presents the characteristic features of the participants—their age, body weight, body height and BMI. The age subgroups accounted for the same proportions as in the general Polish population as was stated according to the Statistical Yearbook [30], the chi-square test revealed no significant differences between typical and observed numerical strength in the analysed group (*p* = 0.2078). Significant age differences were found between groups with normal body weight and obese individuals (*p* = 0.0057)—the normal body weight subjects were younger.

The medians of energy value of the diet in groups with normal body weight, overweight, obese class I (BMIϵ < 30.0; 35.0 kg/m^2^) and obese class II/III individuals (BMI ≥ 35.0 kg/m^2^) were, respectively, 1593 kcal (807–2456 kcal), 1483 kcal (773–2868 kcal), 1421 kcal (359–2652 kcal) and 1343 kcal (646–3065 kcal) (the distribution of this factor in the groups was different than that in the normal) (*p* = 0.0271 for the general difference between the BMI groups). It was concluded that the energy value of the diet differed in groups with normal body weight and obese class II/III individuals (difference of 250 kcal for the comparison of medians; *p* = 0.0278).

Sodium, potassium, calcium, phosphorus, magnesium, iron, zinc and copper intake in the diets as well as intake per 1000 kcal in the analysed group of women, accompanied by the comparison between the BMI groups, is presented in Figs. 1, 2, 3, 4, 5, 6, 7 and 8, respectively.

**Fig. 1:**
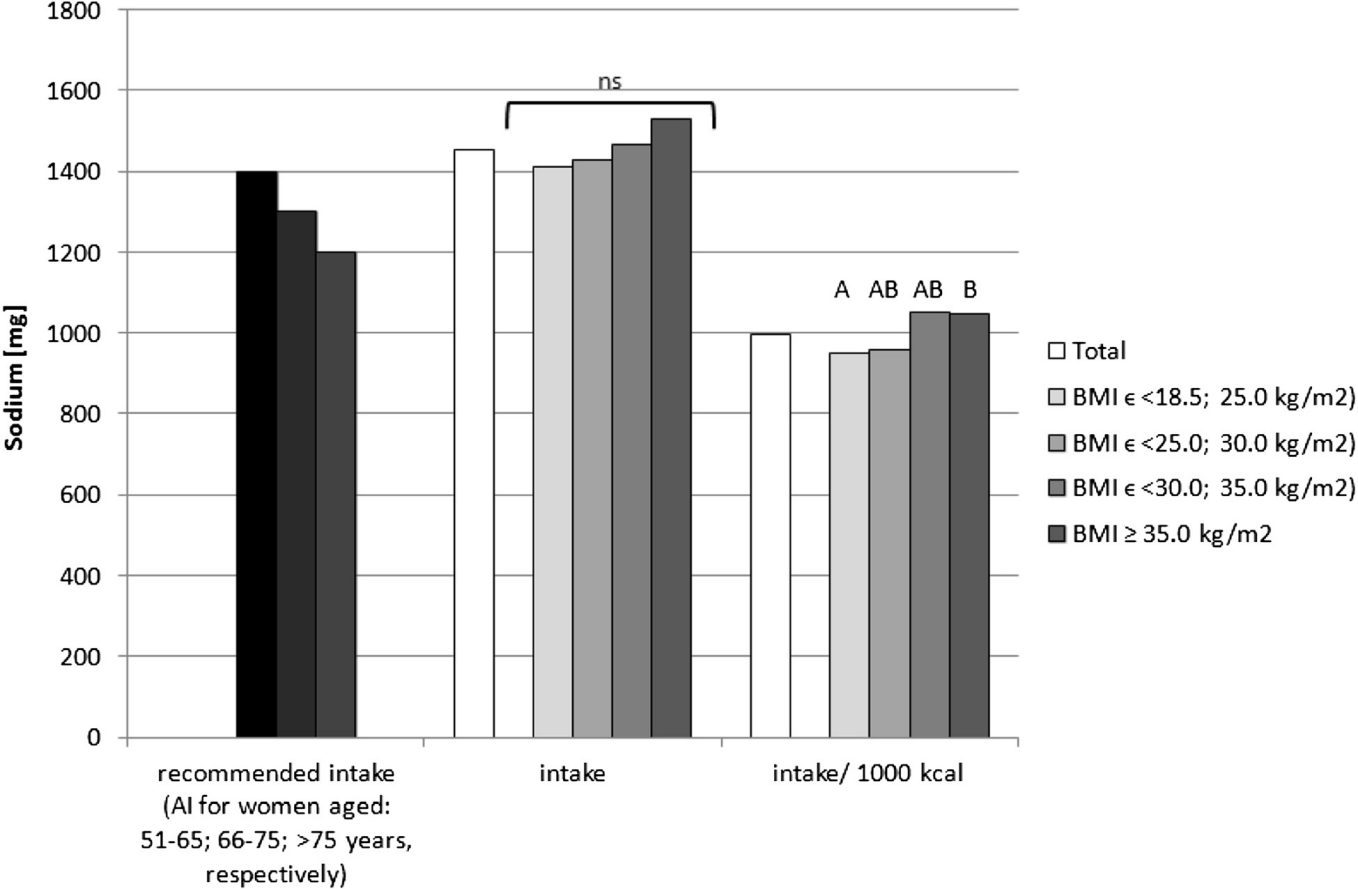
Recommended sodium intake [34], accompanied by comparison of sodium intake (*p* = 0.8574) and of sodium intake per 1000 kcal of diet (*p* = 0.0081) between BMI groups, in analysed healthy women aged >55, RAC-OST-POL study, May 2010. *AI* Adequate Intake level, *ns* not significant differences. *A* and *B* values with unlike letters were significantly different (*p* < 0.05)

**Fig. 2:**
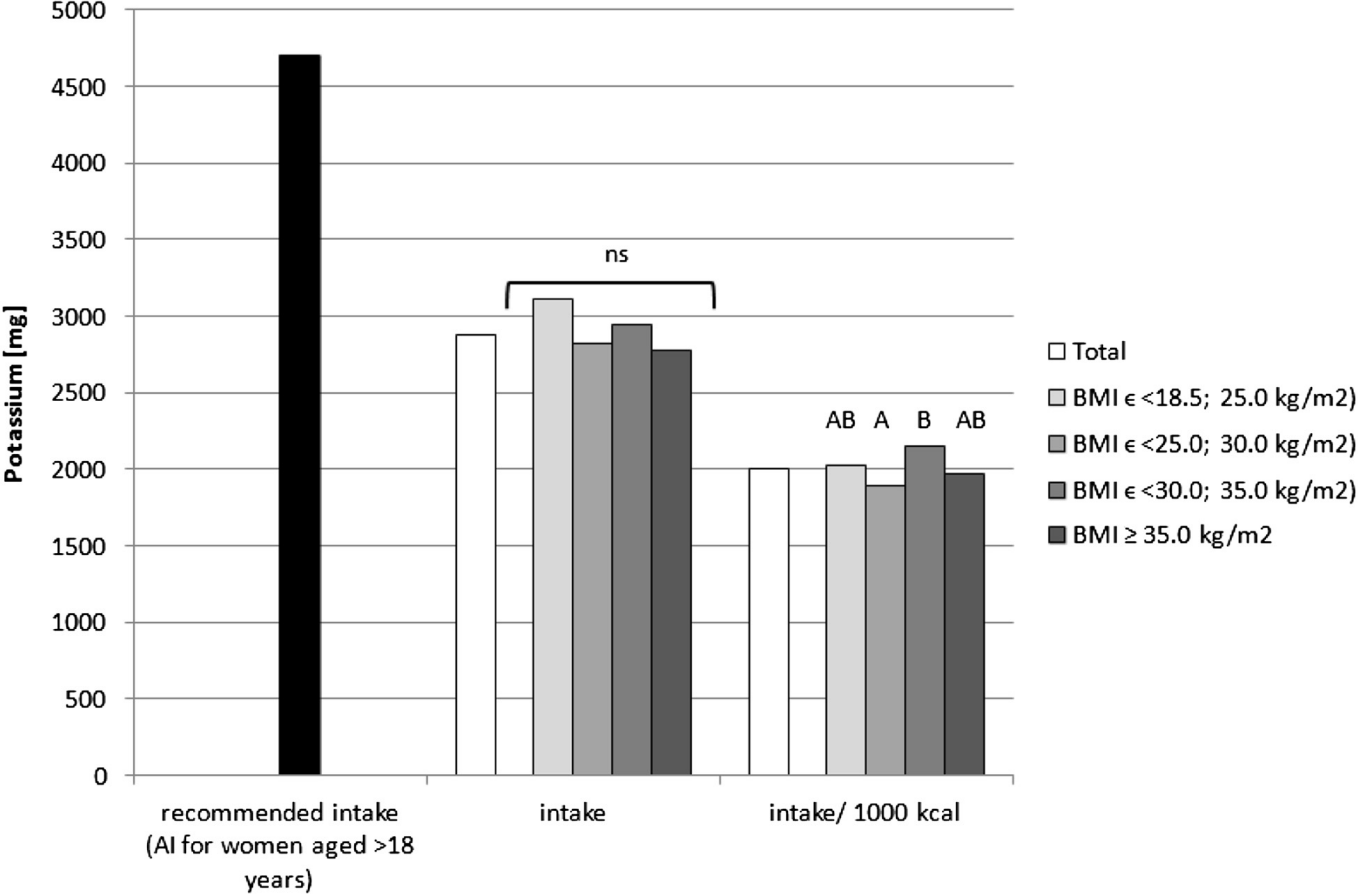
Recommended potassium intake [34], accompanied by comparison of potassium intake (*p* = 0.0726) and of potassium intake per 1000 kcal of diet (*p* = 0.0169) between BMI groups, in analysed healthy women aged >55, RAC-OST-POL study, May 2010. *AI* Adequate Intake level, *ns* not significant differences. *A* and *B* values with unlike letters were significantly different (*p* < 0.05)

**Fig. 3:**
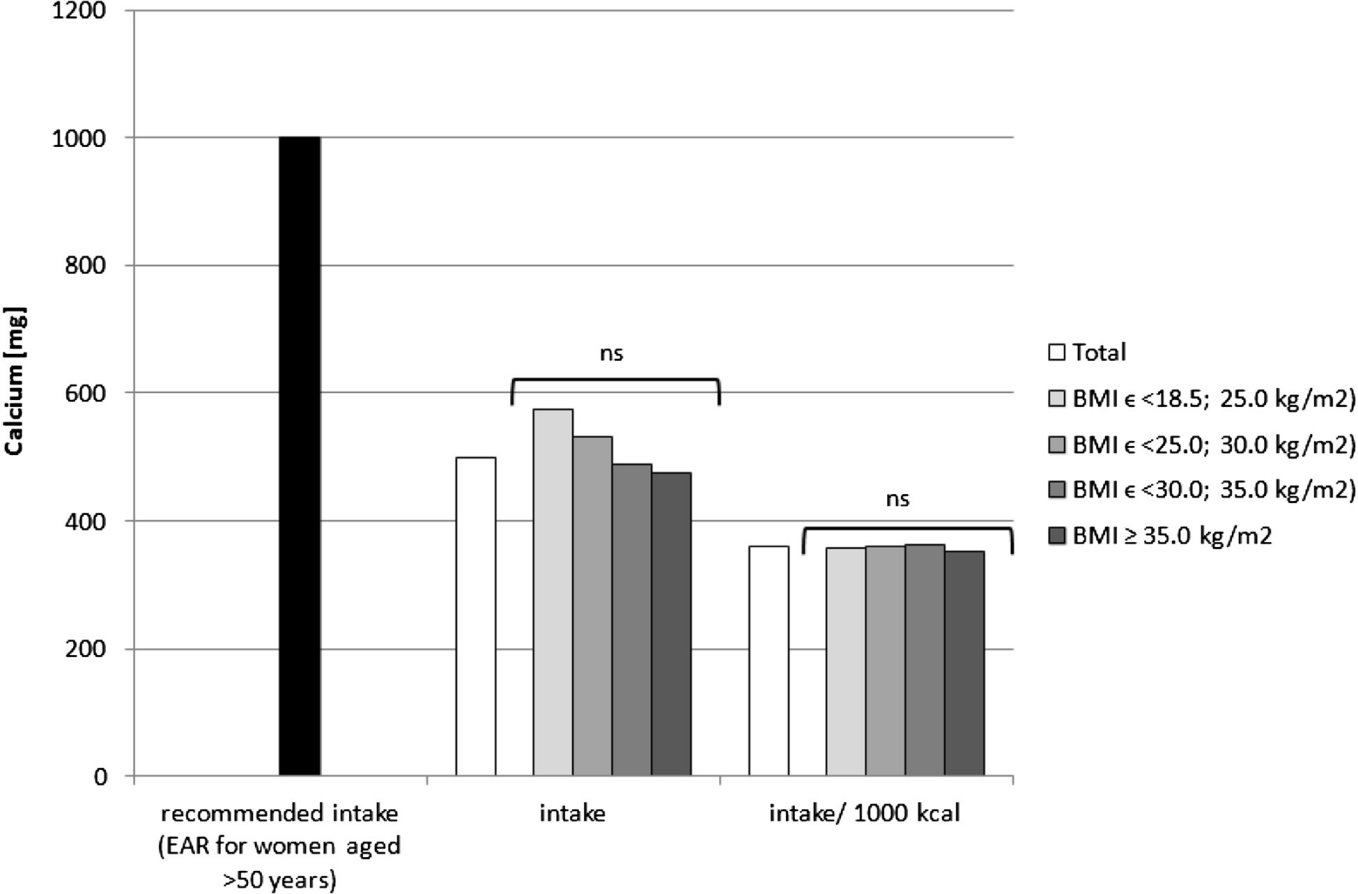
Recommended calcium intake [34], accompanied by comparison of calcium intake (*p* = 0.0619) and of calcium intake per 1000 kcal of diet (*p* = 0.7191) between BMI groups, in analysed healthy women aged >55, RAC-OST-POL study, May 2010. *EAR* Estimated Average Requirement level, *ns* not significant differences

**Fig. 4:**
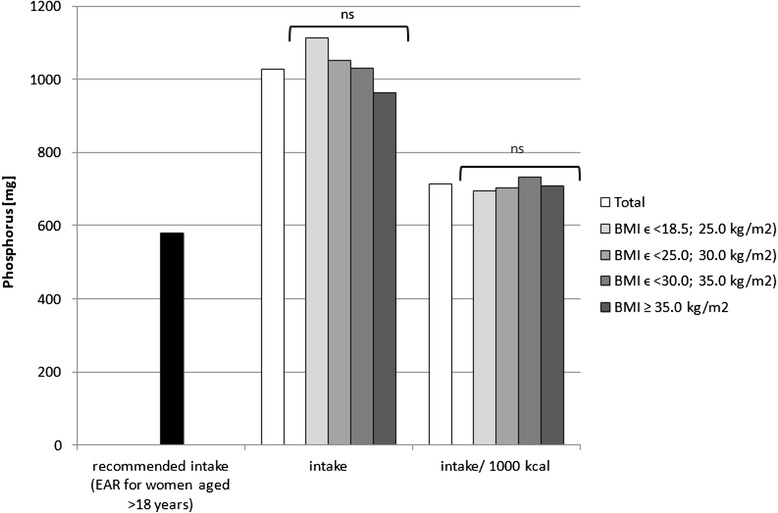
Recommended phosphorus intake [34], accompanied by comparison of phosphorus intake (*p* = 0.0892) and of phosphorus intake per 1000 kcal of diet (*p* = 0.3737) between BMI groups, in analysed healthy women aged >55, RAC-OST-POL study, May 2010. *EAR* Estimated Average Requirement level, *ns* not significant differences

**Fig. 5:**
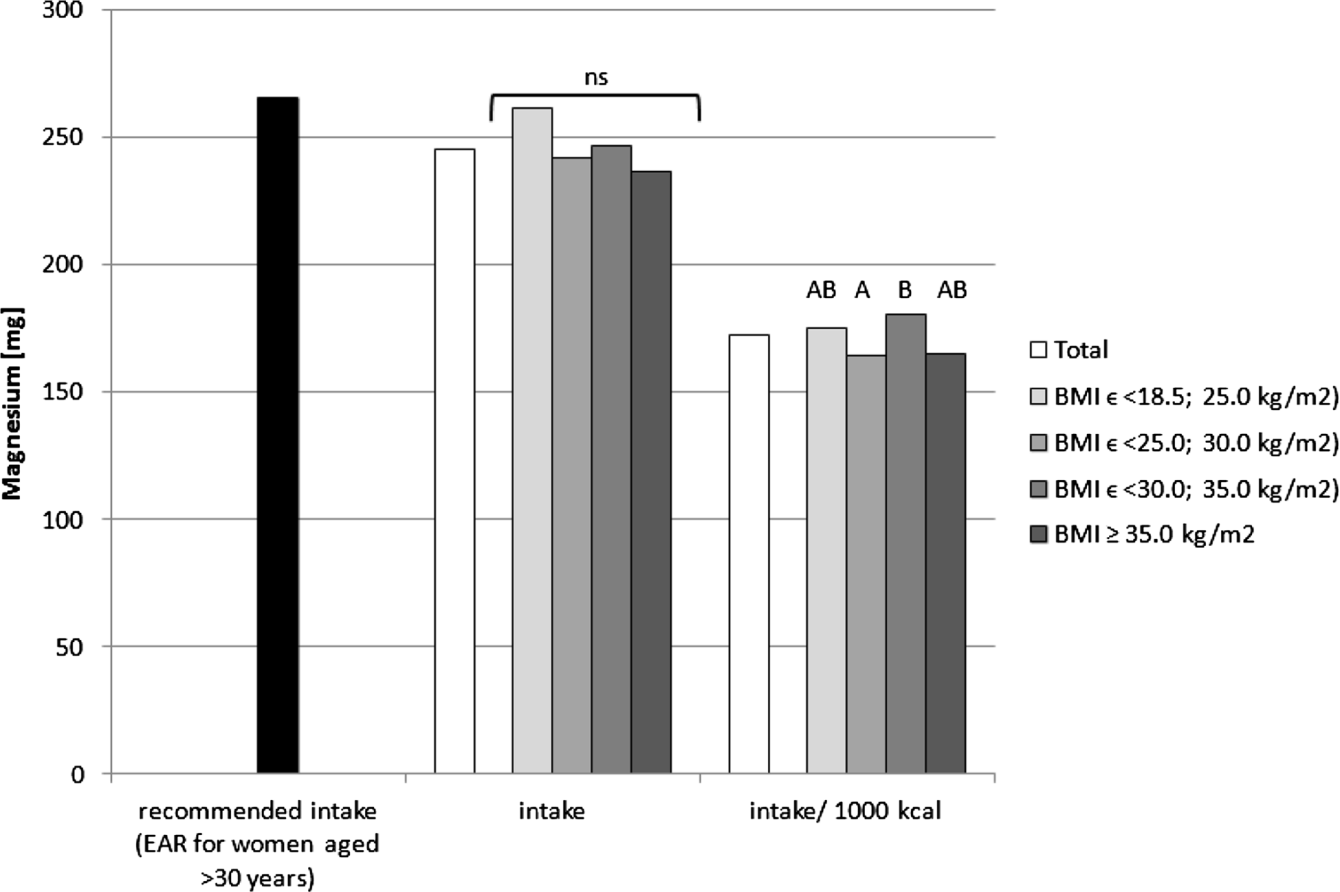
Recommended magnesium intake [34], accompanied by comparison of magnesium intake (*p* = 0.1497) and of magnesium intake per 1000 kcal of diet (*p* = 0.0323) between BMI groups, in analysed healthy women aged >55, RAC-OST-POL study, May 2010. *EAR* Estimated Average Requirement level, *ns* not significant differences. *A* and *B* values with unlike letters were significantly different (*p* < 0.05)

**Fig. 6:**
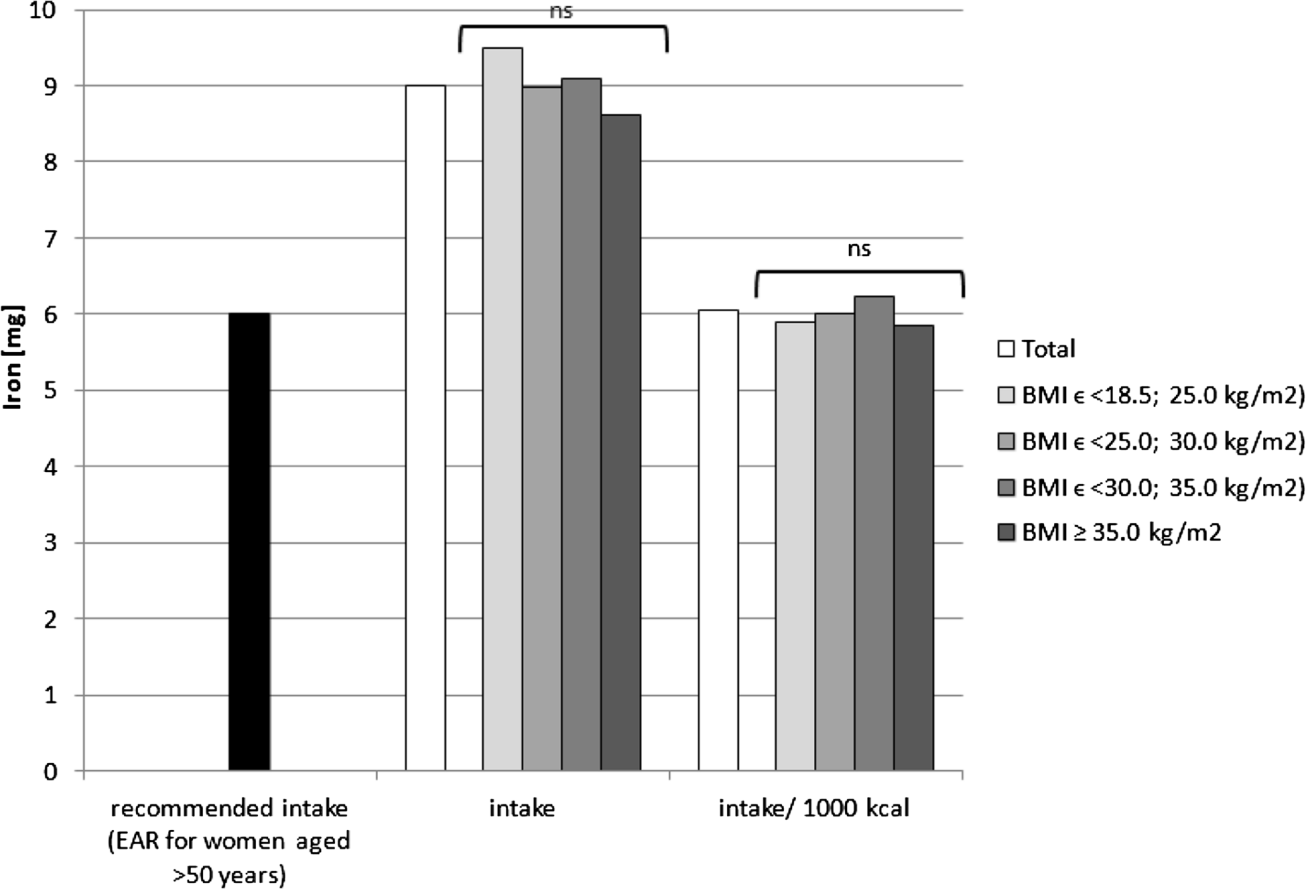
Recommended iron intake [34], accompanied by comparison of iron intake (*p* = 0.5120) and of iron intake per 1000 kcal of diet (*p* = 0.1563) between BMI groups, in analysed healthy women aged >55, RAC-OST-POL study, May 2010. *EAR* Estimated Average Requirement level, *ns* not significant differences

**Fig. 7:**
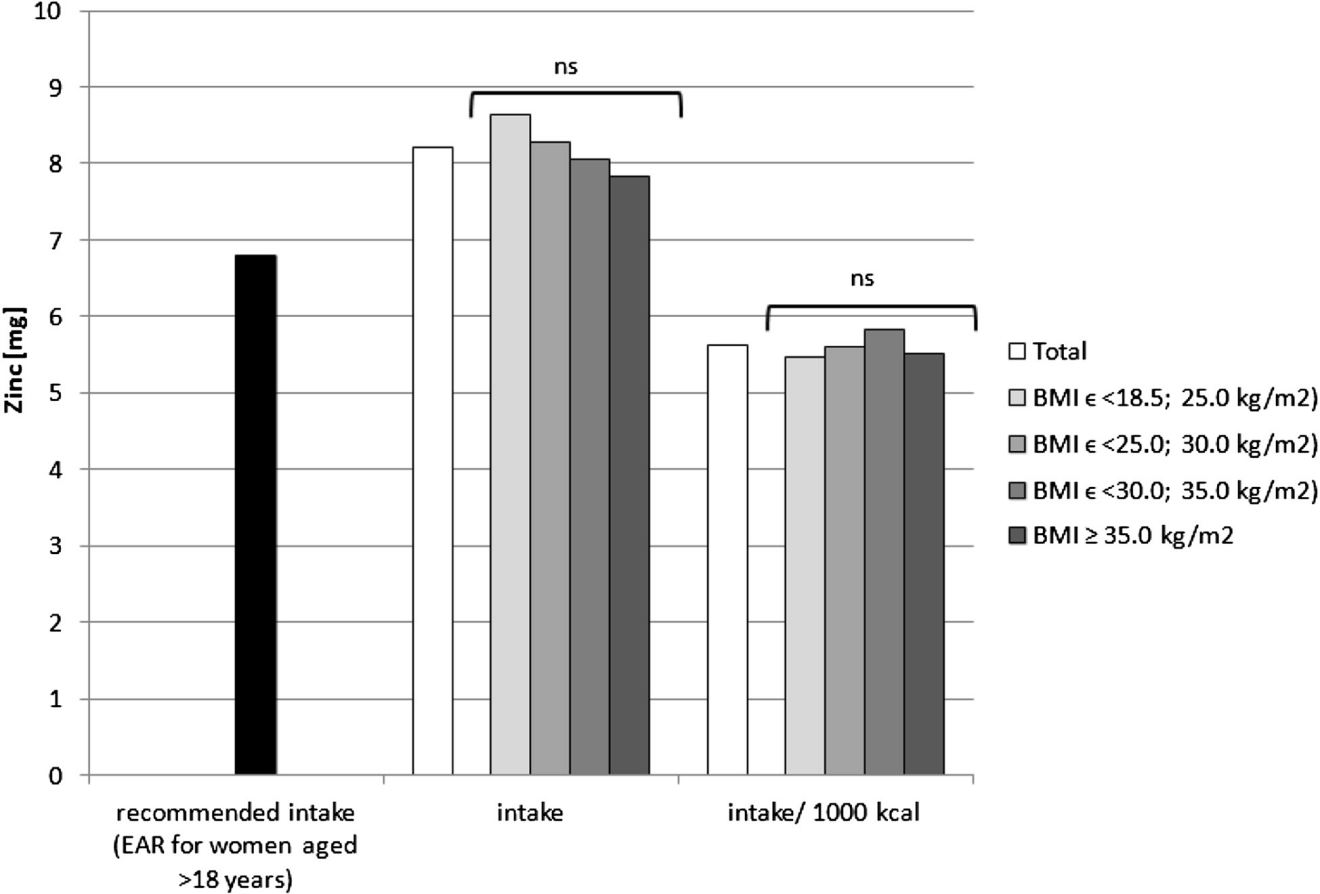
Recommended zinc intake [34], accompanied by comparison of zinc intake (*p* = 0.2771) and of zinc intake per 1000 kcal of diet (*p* = 0.2324) between BMI groups, in analysed healthy women aged >55, RAC-OST-POL study, May 2010. *EAR* Estimated Average Requirement level, *ns* not significant differences

**Fig. 8:**
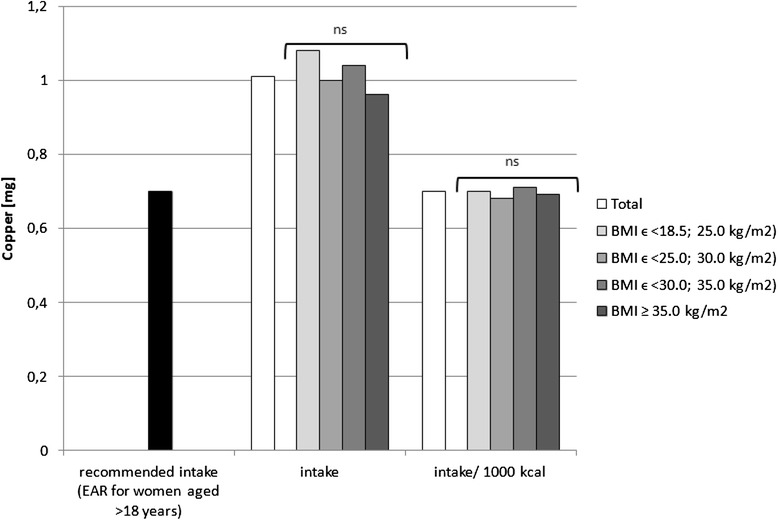
Recommended copper intake [34], accompanied by comparison between BMI groups of copper intake (*p* = 0.1660) and of copper intake per 1000 kcal of diet (*p* = 0.1936) between BMI groups, in analysed healthy women aged >55, RAC-OST-POL study, May 2010. *EAR* Estimated Average Requirement level, *ns* not significant differences

No significant differences were observed in the analysed mineral intake in the diets between the BMI groups. Statistically significant differences of intake per 1000 kcal between groups were observed for sodium (*p* = 0.0081), potassium (*p* = 0.0169) and magnesium (*p* = 0.0323). Individuals with normal body weight were characterised by lower sodium intake per 1000 kcal of diet (about 100 mg/1000 kcal) in comparison with obese class II and III individuals (BMI ≥ 35.0 kg/m^2^). Simultaneously, overweight individuals were characterised by lower potassium (about 250 mg/1000 kcal) and magnesium intake per 1000 kcal of diet (about 20 mg/1000 kcal) in comparison with obese class I individuals (BMIϵ < 30.0; 35.0 kg/m^2^).

The mineral content in the diets in the analysed group of women, in comparison with Polish recommendations for intake of analysed minerals (also presented in the Figs. 1, 2, 3, 4, 5, 6, 7, 8), and accompanied by a comparison of satisfying nutritional needs between the BMI groups, is presented in Table 2. Most of the individuals were characterised by insufficient intake of potassium, calcium and magnesium. No statistically significant differences in satisfying nutritional needs between the BMI groups (*p* > 0.05) were observed for all analysed minerals.

**Table 2:** Mineral content in diets in analysed group of healthy women aged >55, in comparison with recommendations, accompanied by comparison of satisfying nutritional needs between BMI groups, RAC-OST-POL study, May 2010

Group	Number	% of group	Na	K	Ca	P	Mg	Fe	Zn	Cu
Total	405	Below AI/EAR	39.3	96.3	92.1	4.7	61.2	6.2	28.1	10.6
	405	Above AI/EAR	60.7	3.7	7.9	95.3	38.8	93.8	71.9	89.4
BMIϵ <18.5; 25.0 kg/m^2^	61	Below AI/EAR	41.0	95.1	90.2	1.6	50.8	9.8	23.0	8.2
	61	Above AI/EAR	59.0	4.9	9.8	98.4	49.2	90.2	77.0	91.8
BMIϵ <25.0; 30.0 kg/m^2^	140	Below AI/EAR	42.1	99.3	92.9	7.1	65.7	3.6	26.4	8.6
	140	Above AI/EAR	57.9	0.7	7.1	92.9	34.3	96.4	73.6	91.4
BMIϵ <30.0; 35.0 kg/m^2^	118	below AI/EAR	35.6	93.2	92.4	3.4	56.8	5.1	28.0	11.0
	118	Above AI/EAR	64.4	6.8	7.6	96.6	43.2	94.9	72.0	89.0
BMI ≥ 35.0 kg/m^2^	86	Below AI/EAR	38.4	96.5	91.9	4.7	67.4	9.3	34.9	15.1
	86	Above AI/EAR	61.6	3.5	8.1	95.3	32.6	90.7	65.1	84.9
*p* ^a^	*p* ^a^	*p* ^a^	0.7392	0.0751	0.9311	0.3080	0.0956	0.1915	0.3993	0.4156

*Na* sodium, *K* potassium, *Ca* calcium, *P* phosphorus, *Mg* magnesium, *Fe* iron, *Zn* zinc, *Cu* copper, *AI* Adequate Intake level (established for sodium and potassium), *EAR* Estimated Average Requirement level (established for calcium, phosphorus, magnesium, iron, zinc and copper)

^a^Differences assessed by the chi-square test

## Discussion

In the research of Mirmiran et al. [31], the effect of under-reporting of energy intake on estimates of nutrient intake was determined. It was concluded that the under-reporters were characterised by a higher BMI in comparison with the normal-reporters, which was observed for both female and male subjects. Also, in a study by Goris et al. [32], it was stated that under-reporting by obese individuals is commonly observed and is explained by both under-recording and under-eating.

A similar conclusion may be reached in the analysed group when taking into account the significantly higher energy value of the declared diets of individuals with normal body weight than those from obese class II/III. Under-reporting may be considered in the analysed group, as it is in general quite commonly stated in overweight and obese Caucasian female individuals [33] and older individuals [34]—it is more common than in male [33] and younger individuals [34]. The possible reasons for under-reporting among excessive body mass individuals have been stated to be associated with the fact that in Western countries, obesity is perceived as a highly stigmatised condition and obese individuals experience social pressure to reduce weight or even sometimes social discrimination [35].

Since the energy requirement is associated with, inter alia, physical activity and body mass [36], it may be concluded that in the group of female postmenopausal individuals, who were characterised by a similar physical activity level, in stable body mass conditions, individuals characterised by a higher BMI are simultaneously characterised by higher energy intake. Such under-reporting impacts the obtained nutritional value of the diets [37]. On the other hand, another reason for the observed situation may have been over-reporting of the physical activity level, which was observed in overweight female individuals [38].

In the previously mentioned research of Mirmiran et al. [31], the intake of iron, calcium, phosphorus, magnesium and potassium was lower in the under-reporters (who were simultaneously characterised by a higher BMI than that in the normal-reporters); however, following adjustment, no significant differences were observed. In the research presented, the observed under-reporting in the higher BMI individuals also impacted mineral intake; nonetheless, no differences were observed in the case of reported intake but were revealed after adjustment (recalculating the nutritional value per 1000 kcal). It may be concluded that if overweight and obese individuals report a reliable intake, differences in the case of the reported intake (without recalculation) would be observed, as the consumption of food products and, as a consequence, the intake of minerals in overweight and obese individuals is possibly higher than in normal body weight individuals.

Differences in nutrient density (between the nutritional value recalculated per 1000 kcal) were observed in the analysed group for sodium (normal body weight vs. obese class II and III individuals), potassium (overweight vs. obese class I individuals) and magnesium (overweight vs. obese class I individuals). For sodium and potassium, the same conclusions were formulated in a population-based Swiss research study, where the intake of sodium, potassium and protein increased across the BMI categories in both men and women with *p* = 0.001 [39]. Similar observations were made in a Spanish FANPE study, where higher sodium intake was observed among excessive body weight than in normal body weight individuals [40]. Also, in the Olivetti Heart Study conducted in Italy, higher salt intake and altered renal sodium handling were observed in overweight and obese participants [41]. Equally, in the Italian MINISAL-SIIA study on hypertensive individuals, being overweight and obesity were associated with particularly high sodium intakes [42]. Despite the fact that in the research presented, sodium chloride addition during meal preparation was not taken into account, the presented results may be of great value as they result from natural sodium content in the products and sodium content in processed products chosen by the analysed subjects.

The higher sodium intake that was observed in individuals characterised by higher BMI may result in higher blood pressure, as sodium intake is a well-known factor of hypertension development [43]. However, the results of Baudrand et al. [44] revealed that a high sodium diet is associated not only with hypertension but also with insulin resistance, dyslipidaemia and hypoadiponectinaemia, even when adjusting by confounding variables. Also, in the research of Aaron et al. [45], it was concluded that in obese adults, higher dietary sodium intakes were associated with albuminuria. As a consequence, high sodium intake is an additive mechanism in obesity-related metabolic disorders.

On the other hand, the higher potassium intake that was observed in the analysed group of obese individuals in comparison with overweight individuals is, according to the fourth Korean National Health and Nutrition Examination Survey (KNHANES IV), inversely associated with metabolic syndrome in adults [46]. This may result from the fact that a low serum potassium level is significantly associated with the prevalence of metabolic syndrome [47]. Taking into account the fact that potassium intake increases, similarly as sodium intake, across BMI categories, both in the research presented and in that of Ogna et al. [39], it may be the factor that reduces the potential frequency of obesity-related metabolic disorders in overweight and obese individuals.

Simultaneously, the higher magnesium intake that was observed in the analysed group for obese individuals in comparison with overweight individuals was also, according to the results obtained by other authors, associated with lower insulin resistance [48]. In the OLETF rat model (a model of diabetes type II with obesity), under conditions of excessive food intake, magnesium supplementation even delayed the development of diabetes [49]. Also, the meta-analysis of prospective cohort studies revealed that magnesium intake is significantly inversely associated with the risk of type 2 diabetes in a dose-response manner [50]. Similarly, as in the case of potassium, magnesium intake, which is higher in obese than in overweight individuals, may be the factor reducing the potential frequency of diabetes, which is one of the obesity-related metabolic disorders in overweight and obese individuals.

Potassium, calcium and magnesium are well-known factors associated, inter alia, with cardiovascular function [51], bone health [52] and neurological transmission [53]. Their deficiency may also be related to cancer development [54]. The diets of the analysed individuals cannot be defined as properly balanced, based on the low intake of potassium, calcium and magnesium—which were lower than the recommended level. Even if obese women have higher intake of potassium and magnesium than overweight women, in each group, over 90 % of the subjects declared insufficient calcium and potassium intake, and over 50 % declared insufficient magnesium intake. Moreover, it should be indicated that the absorption of the above-mentioned nutrients was not analysed, whereas it may also be influenced by vitamin intake, e.g. vitamin D intake may influence calcium [55] and magnesium absorption [56] or vitamins A and B may influence iron absorption [57].

Following an improperly balanced diet, especially if it is constant, may have serious medical consequences, as were mentioned previously (e.g. diabetes, hypertension, cardiovascular diseases); thus, nutritional education should be instituted in all BMI groups, as it is stated to be effective in improving the nutritional status and nutritional value of the diets of elderly patients [58]. Even if it is supposed that overweight and obese women under-reported consumption and the actual nutritional value of their diets was different (their mineral intakes were probably also higher), they need nutritional education to adjust the amount of consumed food products to their actual requirements and body mass.

In general, it may be stated that overweight and obese postmenopausal women probably under-report the amount of consumed food products, which influences the declared mineral intake. Sodium intake, which is higher for obese than for normal body weight female individuals, seems to be typical in population studies and may be an additional element contributing to metabolic syndrome development. Simultaneously, the higher nutrient density that was observed for potassium and magnesium in female postmenopausal individuals characterised by a higher BMI may partly counterbalance the negative impact of higher sodium intake.

## Conclusions

In the analysed group of postmenopausal female individuals, it was observed that they were following an improperly balanced diet. It was stated that daily intake of all assessed minerals was not BMI-dependent for postmenopausal female individuals, but the nutrient density of the diet (for sodium, potassium and magnesium) was associated with BMI. It may be concluded that in the analysed group of postmenopausal female individuals, following an improperly balanced diet may contribute to a variety of negative health consequences and should be overcome by nutritional education.
